# Effects of Zolpidem on Sleep Quality and Quality of Life in Stroke Patients with Comorbid Depression

**DOI:** 10.1192/j.eurpsy.2026.11074

**Published:** 2026-07-31

**Authors:** A. Starcevic

**Affiliations:** Neuroanatomy, University of Belgrade, Belgrade, Serbia

## Abstract

**Introduction:**

Post-stroke insomnia and depression are prevalent, negatively impacting rehabilitation and quality of life (QoL). Zolpidem, a non-benzodiazepine hypnotic, is commonly used for insomnia but its effects in stroke patients with comorbid depression remain underexplored.

**Objectives:**

This study investigates the efficacy and safety of zolpidem in improving sleep quality and QoL in this population.

**Methods:**

In a randomized, double-blind, placebo-controlled trial, 60 post-acute ischemic stroke patients, both genders, (aged 45–75 years) with DSM-5 diagnosed insomnia and comorbid depression were assigned to receive either zolpidem (10 mg) or placebo nightly for 4 weeks. Sleep quality was assessed using the Pittsburgh Sleep Quality Index (PSQI) and Insomnia Severity Index (ISI). QoL was evaluated using the Short Form Health Survey (SF-36). Secondary outcomes included depression severity (Hamilton Depression Rating Scale, HAM-D) and adverse events. Assessments were conducted at baseline and after four weeks.

**Results:**

Zolpidem significantly improved sleep quality compared to placebo, with a mean PSQI score reduction of 4.8 ± 1.2 vs. 1.9 ± 0.8 (p < 0.001) and ISI score reduction of 6.2 ± 1.5 vs. 2.3 ± 1.0 (p < 0.01). SF-36 scores showed greater improvement in physical (p = 0.02) and mental (p = 0.01) QoL domains in the zolpidem group. HAM-D scores decreased significantly in the zolpidem group (p = 0.03), suggesting a potential indirect effect on depression. Side effects (dizziness, daytime drowsiness) were mild and occurred in 10% of the zolpidem group vs. 5% of the placebo group, with no serious side effects reported.

**Conclusions:**

Zolpidem is effective in improving sleep quality and QoL in stroke patients with comorbid depression, with a favorable safety profile. These findings support its potential as a therapeutic option in this population, warranting further investigation into long-term effects and optimal dosing strategies.

**Keywords:**

Zolpidem, insomnia, stroke, depression, quality of life, sleep quality

**Disclosure of Interest:**

None Declared

